# Novel strategies of protecting non-cancer cells during chemotherapy: Are they ready for clinical testing?

**DOI:** 10.18632/oncotarget.249

**Published:** 2011-03-29

**Authors:** Zbigniew Darzynkiewicz

**Affiliations:** ^1^ Brander Cancer Research Institute and Department of Pathology, New York Medical College, Valhalla, NY, 10595

Chemotherapy and radiotherapy are the most effective anticancer modalities. However the effectiveness of chemotherapy, which targets DNA replication and mitosis, is limited by the side effects resulting from killing normal cells that proliferate. The discovery of molecular abnormities associated with neoplastic growth stimulated attempts to develop *targeted therapies* aimed at selectively inhibiting growth of cancer by interfering with specific molecules or signal transduction pathways that are over-expressed (overactive) in cancer cells. It was anticipated that targeted cancer therapies would be more effective than current treatments and *less harmful to normal cells* [1]. Imatinib mesylate (Gleevec, STI–571), the drug developed to treat Philadelphia chromosome positive CML, exemplifies the first successful application of targeted therapy [2].The success, however, is due to the fact that the target (Bcr-Abl tyrosine kinase) fulfils two criteria: (i) is unique to this leukemia and not present in normal cells, and (ii) is essential for cell growth. Most other tumors do not express such unique features and therefore targeted treatments, especially of patients with most common solid tumors, has been less successful and is frequently disappointing [3]. This is not surprising because the target is usually the component of a cell signaling pathway that, while over-expressed in cancer, is essential for the survival of cancer *as well as normal cells*. For example, if a cancer cell has e.g. 10^7^ - and normal cell 10^3^ – such cell signaling molecules, the treatment with the targeting inhibitor that inactivates 99.99% of these molecules will completely deplete the normal cells but leave the cancer cells still with 10^3^ molecules. Thus, although the initial cancer response to the inhibitor may be quite dramatic, the escalation of dose and treatment duration is expected to lead to preferential killing of normal rather than cancer cells. Given the above, chemo- and radiotherapy are likely to remain the major therapeutic approaches in the armamentarium of oncologist for the near future.

As mentioned, the side effects of chemotherapy limit its effectiveness. To overcome this limitation strategies designed to protect non-cancer cells during chemotherapy have been proposed. Since chemotherapeutic drugs target DNA replication (DNA damaging agents) or mitosis (mitotic blockers), strategies were aimed to combine chemotherapy with inhibitors that would halt progression of normal cells through the cell cycle, making them drug-resistant during the duration of their arrest [3-6]. In most cancers the regulation of cell cycle progression is impaired, especially as a result of dysfunctional p53 or retinoblastoma pathway. Therefore, the inhibitors targeting these pathways cannot effectively stop the cell cycle of cancer cells and therefore cannot offer protection to cancer cells. While such strategies, defined as cyclotherapy [5,6]. have obvious rationale, their implementation in the clinic was difficult, being delayed by the need for extensive clinical trials of the cell cycle inhibitors (e.g. inhibitors of Cdks) as some of them may show toxicity. Furthermore, a highly balanced equilibrium between the protective- versus chemotherapeutic- agents, both in terms of their respective concentrations as well as the sequence of and length of administration, must be tested to achieve successful clinical application.

In the current article in *Oncotarget,* Apontes *et al* [7] describe advances that move forward the potential clinical applications of cyclotherapy. The authors used mitotic inhibitors, paclitaxel and nocodazole as the chemotherapeutic agents designed to kill cancer cells. The strategy of normal cells protection relied on the use of either non-genotoxic inducer of p53, nutlin-3a (N-3a), the inhibitor of mTOR pathway rapamycin (RAPA), or the widely prescribed anti-diabetic drug, possibly affecting IGF-1 signaling, metformin (MF), each tested alone and in combination. Their data are very encouraging. Specifically, the authors have seen that N-3a, RAPA or MF, particularly when applied in combinations, halted cell cycle progression of the three normal human cell lines cells, arresting them reversibly in G_1_ and/or G_2_ and thereby protecting from the toxicity of mitotic inhibitors. No such arrest was observed in the case of breast cancer MDA-MB-231 cells having mutant p53. Of importance was the observation that the arrest of normal cells was achieved: (i) for the duration equivalent of the time interval during which the treatment of cancer cells with mitotic inhibitors (3 days) eliminated their capability to proliferate (assessed 6 days later); and (ii) the arrest was to a large degree reversible and showed no evident toxicity. The maximal protective effects were seen in drug combinations such as N-3a+RAPA, N-3a+RAPA+MF, or RAPA+MF. Of further interest was the observation that while the protective effect of RAPA+MF for normal cells was seen at the reduced concentration of glucose such conditions were actually cytotoxic for cancer cells. The authors offer specific recommendations on timing and sequence of administration of protective agents versus mitotic inhibitors in treatment of cancer.

Several implications advancing clinical use of cyclotherapy stem from the findings of Apontes *et al.*^7^ One is that the protective agents MF and RAPA, albeit for different applications, are already widely used in the clinic. Therefore their toxicity and pharmacokinetics are well characterized. N-3a, while not advanced in clinical trials, appears to be mimicked in terms of its ability to activate p53, by low concentrations of actinomycin D [6,8], the drug whose clinical application is also recognized. The agents that can be used to protect normal cells thus have been already clinically tested. Another observation of clinical importance stems from the use of metformin. While metformin was shown to have the property to protect normal cells it also has antineoplastic activity against prostate,[9] breast [10] and other cancers [11]. Application of MF+RAPA, perhaps combined with fasting [12] or with 2-deoxyglucose [12] (2-DG) to reduce glucose utilization (2-DG is also well characterized for clinical use) may provide concurrently the protection of normal cells and the antineoplastic activity.

The advantage of cyclotherapy stems from the fact that response of normal cells to the protective agents targeting their signaling and metabolic pathways, which are well characterized, is predictable. In contrast, targeting cancer is uncertain since many of the pathways, often different in various cancers, are dysfunctional. Furthermore, even personalized targeting, after identification of the defective pathways of a given cancer, may be inadequate because of changes that may occur due to tumor progression, in the time interval between tumor sampling and its full characterization.
